# Depressor Labii Inferioris Muscle Palsy After Chin Augmentation

**DOI:** 10.7759/cureus.73309

**Published:** 2024-11-08

**Authors:** Ahmet Volkan Kurtoglu, Sule Ozturk Kurtoglu, Zeliha Matur

**Affiliations:** 1 Department of Neurology, Bezmialem Vakif University, Faculty of Medicine, Istanbul, TUR; 2 Department of Pediatrics, Istanbul Medipol University, Faculty of Medicine, Istanbul, TUR

**Keywords:** chin augmentation, depressor labii inferioris muscle, marginal mandibular nerve, needle electromyography, surgical complication

## Abstract

The depressor labii inferioris (DLI) muscle helps to lower and turn the lower lip outward and receives innervation from the marginal mandibular branch of the facial nerve. Paralysis of this muscle is a rare but potential complication of chin augmentation injections. Paralysis of the DLI causes symptoms such as difficulty smiling on the affected side, difficulty speaking clearly, and facial asymmetry, especially when smiling. In this case report, we present two female patients, aged 29 and 36 years, who were evaluated for impaired lower lip movement, asymmetry in smiling, and slurred speech following submandibular liposuction and chin augmentation. The patients were found to have DLI palsy, closely followed with facial exercises, and recovered completely within three months. Although isolated DLI palsy is rare, it may occur as a complication of various maxillofacial surgeries. Careful technique and good knowledge of facial anatomy are important to prevent complications.

## Introduction

Smiling is one of the most frequently used facial expressions in human social life. This expression requires the lips to perform several movements simultaneously. One component of the smile is the depression of the lower lip. The main muscles responsible for this movement are the depressor labii inferioris (DLI) and depressor anguli oris, innervated by the marginal mandibular nerve (MMN), a branch of the facial nerve [1].

Iatrogenic isolated MMN injury is considered rare; however, due to its relatively long and superficial anatomical course, it may manifest as a complication of cervical dissection, carotid endarterectomy, thyroidectomy, or various surgical procedures involving the cervical and submandibular regions [2,3]. Although it is known that it can also occur after aesthetic procedures, there is no comprehensive information in the literature on the presentation and course, except for a few case reports. In this article, we report two cases of involvement of the MMN branches innervating the DLI muscles following aesthetic jaw surgery, along with their clinical course.

## Case presentation

Case 1

**Figure 1:**
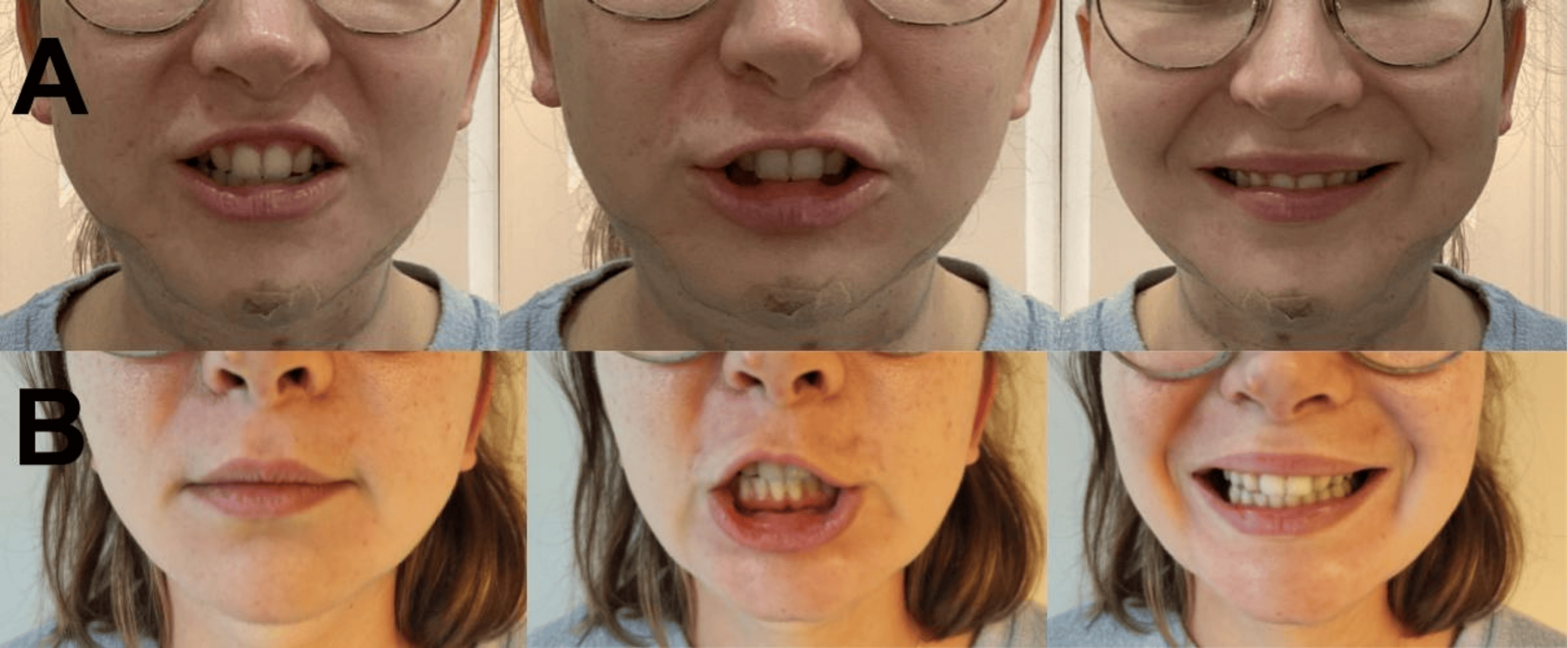
Examination findings of Case 1 A: restricted bilateral downward movement of the lower lip, prominent on the left; while eversion is normal; B: complete recovery of lip movements after treatment

A 29-year-old woman presented with complaints of not being able to show her lower left teeth and slurred speech. She stated that her complaints started after liposuction from the submandibular region and filling the material into the chin. Her medical and family histories were unremarkable. Neurological examination revealed minimal dysarthric speech. She was unable to move the left half of her lower lip downward, whereas she could partially complete the movement of the right half. There was also marked edema around the chin, a surgical scar approximately on the lateral parts of the mental muscles, and ecchymosis in the submental area (Figure 1A). Pre-procedural biochemical evaluations, hepatitis, and HIV serologies had been conducted, and the results were normal. On day 12 of her symptoms, nerve conduction studies (NCSs) showed that the bilateral facial motor response amplitudes recorded from the m. nasalis and m. mentalis were symmetrically normal. Needle electromyography (EMG) revealed acute-phase involvement findings in the MMN fibers innervating the DLI, with complete axonal loss on the left side and near-complete severe axonal loss on the right (Table 1). Follow-up was planned with facial physiotherapy. She was instructed to make a pouting face to draw the lower lip outward and downward, hold this position for five to ten seconds, and then relax. It was recommended that this exercise be repeated ten times daily in front of a mirror. Complete bilateral recovery was observed in three months, more rapidly on the right side (Figure 1B). In parallel with the clinical course, complete reinnervation was observed on EMG follow-up on the right side on day 40 and on the left side on day 83 (Table 1). 

**Table 1:** Needle electromyography findings of the cases FP: fibrillation potentials; MUP: motor unit potentials; N: normal; NE: not examined; PS: positive spikes; L: left; R: right

Muscle	Side	Case 1			Case 2
		12^th^ day	40^th^ day	83^rd^ day	60^th^ day
Depressor labii inferioris	R	Pathologic spontaneous activity (PD, FP) and polyphasic MUPs with decreased recruitment	Polyphasic MUPs with decreased recruitment	N	Pathologic spontaneous activity (PD, FP); long-duration polyphasic MUPs with decreased recruitment
	L	Pathologic spontaneous activity (PD, FP); no MUPs	Polyphasic MUPs with decreased recruitment	Polyphasic MUPs	N
Depressor anguli oris	R	N	N	N	N
	L	N	N	N	N
Mentalis	R	N	N	N	N
	L	N	N	N	N
Orbicularis oris	R	N	NE	NE	N
	L	N	NE	NE	N
Risorius	R	NE	NE	NE	N
	L	NE	NE	NE	N

Case 2 

**Figure 2:**
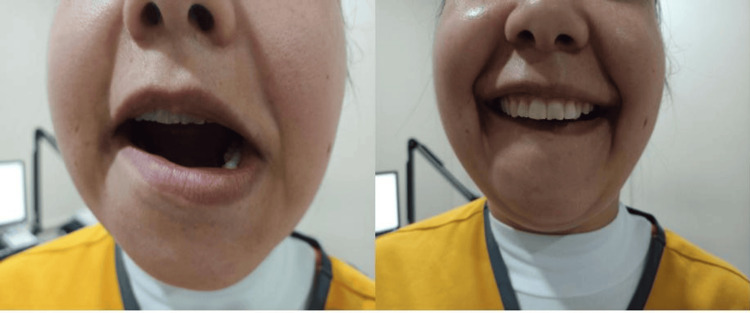
Examination findings of Case 2 Restricted downward movement of the right half of the lower lip.

A 36-year-old woman presented with impaired movement of the right lower lip and an asymmetrical smile. She stated that her symptoms started after a bichectomy and jaw augmentation performed two months ago. Her past medical and family history was unremarkable. Examination revealed mild dysarthria and an inability to move the right half of the lower lip downward (Figure 2). Pre-procedural biochemical evaluations, hepatitis, and HIV serologies had been conducted, and the results were normal. On day 60 of her symptoms, NCSs showed that the bilateral facial motor response amplitudes recorded from the m. nasalis and m. mentalis were symmetrically normal. Needle EMG revealed subacute-phase involvement findings in the MMN fibers innervating the DLI, with near-complete severe axonal loss on the right side (Table 1). She was followed up with facial exercises. Her clinical condition exhibited complete improvement within two months.

## Discussion

There are three primary muscles responsible for the downward movement of the lower lip. Of these, the depressor anguli oris and DLI are innervated by the marginal mandibular branch of the facial nerve, while the platysma is innervated by the cervical branches of the facial nerve [1]. The MMN is one of the terminal branches of the mandibular division of the facial nerve. It arises from the anteroinferior border of the parotid gland, curves toward the submandibular gland, and runs along the angle and inferior border of the mandible below the platysma. Along its course, it innervates the DLI, depressor anguli oris, mentalis, and part of the orbicularis oris. It may anastomose with the buccal and mental nerves on the same side and, more rarely, with the contralateral MMN [4]. The patients presented in this report received chin augmentation using autologous fat as filler material. In the first patient, fat harvested from the neck was utilized, whereas in the second, fat obtained from the cheek was applied. The occurrence of DLI palsy was attributed to the impact of the filler on the marginal mandibular branch of the facial nerve, which innervates this muscle.

In MMN paralysis, the involvement of the depressor muscles limits the eversion, downward, and lateral movements of the lower lip on the affected side, causing asymmetry, especially while smiling. On the affected side, the lower lip may appear loose and slightly inverted, and the teeth may be less visible. It is important to know that the symmetry of the nasolabial sulcus and the movements of the upper lip are preserved in patients with MMN involvement, as it can be confused with central-type facial palsy, especially after carotid endarterectomy in patients with risk factors [5]. In addition, the platysma muscle, which is innervated by the cervical branches of the facial nerve, also contributes to the depression of the lower lip. For this reason, damage to these branches or to the platysma may result in the so-called MMN pseudo-paralysis, which looks similar to MMN damage; however, in this case, the lower lip can be everted due to the preserved function of the mental muscle [6].

MMN involvement may rarely be a component of a syndrome, such as first branchial arch syndrome, or due to isolated congenital causes or local infections, but it is mostly iatrogenic. Major surgical procedures that can cause MMN injury include parotid and mandibular gland surgery, head and neck surgery, carotid endarterectomy, jaw and neck aesthetic procedures, trauma, and orthodontic procedures [3,6,7]. According to various studies in the literature, MMN paralysis can be detected as a surgical complication with rates ranging from 0-29%, depending on the size and type of surgery [2,8]. This condition is not expected to cause severe functional impairment, but it can cause facial asymmetry that can cosmetically affect the person's social life and self-confidence; therefore, follow-up and treatment planning are important, but there is no algorithm for deciding between treatment options. There are several treatment options, such as facial exercises, botulinum toxin application to the contralateral muscles for asymmetry, resection of the depressor muscles on the same side, m. digastricus, or m. extensor digitorum brevis transfer [6,9].

In the present cases, there was no weakness except for the depressor movement of the lower lip. Needle EMG showed findings consistent with axonal damage only in the DLI, supporting these examination findings; however, no pathological findings were found in the other muscles innervated by the MMN, namely the depressor anguli oris, mentalis, and orbicularis oris. In other words, there was an isolated DLI muscle paralysis. Paralysis of the DLI is a rare but potential complication of chin filler injections. It can be caused by inadvertent trauma to the MMN that innervates the DLI during injection. This can happen if the filler is injected too deep or too close to the nerve pathways. Or, an excessive amount of filler or improper placement can cause temporary compression of the nerve, resulting in muscle weakness or paralysis. In rare cases, the filler can also interfere with blood flow, causing ischemia of the nerve or muscle. In most cases of nerve paralysis due to compression or trauma, as in our case, the condition is temporary and recovery is seen within weeks to months due to the very short distance between the nerve injury site and the affected muscle. Gentle massage and facial exercises can help improve muscle function and symmetry. Therefore, the patient was followed with facial exercises, with complete bilateral recovery within three months.

## Conclusions

Paralysis of DLI muscle, though rare, can result in functional and aesthetic impairments, such as facial asymmetry and difficulty smiling. A good knowledge of facial anatomy and correct injection techniques, avoiding deep or excessive injection in areas close to the mandibular branch of the facial nerve, and preventing pressure on surrounding structures by using appropriate filling volumes will reduce this complication.
